# A Novel GMM-Based Behavioral Modeling Approach for Smartwatch-Based Driver Authentication

**DOI:** 10.3390/s18041007

**Published:** 2018-03-28

**Authors:** Ching-Han Yang, Chin-Chun Chang, Deron Liang

**Affiliations:** 1 Department of Computer Science and Information Engineering, National Central University, Taoyuan City 32001, Taiwan, drliang@csie.ncu.edu.tw; 2 Department of Computer Science and Engineering, National Taiwan Ocean University, Keelung City 20224, Taiwan, cvml@mail.ntou.edu.tw; 3 Software Research Center, National Central University, Taoyuan City 32001, Taiwan

**Keywords:** accelerometer sensor, driver authentication, Gaussian mixture models, orientation sensor, smartwatch

## Abstract

All drivers have their own distinct driving habits, and usually hold and operate the steering wheel differently in different driving scenarios. In this study, we proposed a novel Gaussian mixture model (GMM)-based method that can improve the traditional GMM in modeling driving behavior. This new method can be applied to build a better driver authentication system based on the accelerometer and orientation sensor of a smartwatch. To demonstrate the feasibility of the proposed method, we created an experimental system that analyzes driving behavior using the built-in sensors of a smartwatch. The experimental results for driver authentication—an equal error rate (EER) of 4.62% in the simulated environment and an EER of 7.86% in the real-traffic environment—confirm the feasibility of this approach.

## 1. Introduction

Driving behavior differs among drivers. Each driver has a habitual and distinctive driving style; some drive slowly and carefully, while others drive fast and aggressively. Drivers exhibit distinct methods for holding and operating the steering wheel, which differ depending on the driving scenarios (e.g., driving straight, turning, and parking). Recently, several techniques of modeling the behavior of a driver based on the pattern of operating the steering wheel and pedals [1,2,3,4], sitting posture [5], and handgrip patterns [6], have been proposed as methods for driver identification and authentication.

Smartwatches have gained popularity because of technological advances. According to the Gartner report [7], a 17% increase in smartwatch shipments is forecasted for 2018, compared to the 41.5 million units in 2017, and the quantity of yearly shipments is expected to reach nearly 81 million units in 2021. Smartwatches equipped with multiple sensors, such as accelerometers and orientation sensors, can be used not only for health monitoring but also for continuous motion analysis [8]. These devices have been utilized for many applications, such as gesture detection [9,10], health monitoring [11,12,13], information security [14,15], personal safety [16,17], and other applications [18].

Recently, smartwatches have been used to analyze the behavior of a driver for user authentication. Liang and Kotz [19] developed a smartwatch-based user-presence authentication system that continuously authenticated the user with a computer. This system required the hand- and mouse-motion data of the user, and matched the enrolled pattern through three steps: peak detection, weight calculation, and distance calculation. Lewis et al. [20] developed a gesture-based real-time authentication system for a smartwatch. Their system applied behavioral biometrics collected from the readings of the accelerometer and gyroscope of a smartwatch, and used the dynamic time warping algorithm for template generation and matching. These two studies on smartwatches show that the characteristics of a user’s hand movement can be analyzed through the built-in sensor of a smartwatch. On the other hand, Lee et al. [21] developed a real-time driver vigilance monitoring system that tracked a user’s steering wheel movement (SWM) through the motion sensor of a smartwatch and the heart rate from a photoplethysmogram (PPG) sensor. Pearson’s method was applied to select time, phase, and frequency domain features extracted from the SWM, PPG, and PPG-derived respiration. Driver hypervigilance was estimated according to the result of the weighted fuzzy c-mean algorithm. Lee et al. [22] detected a driver’s drowsiness by the time, spectral, and phase domain features of the driver’s hand movements. The accelerometer and gyroscope of a smartwatch captured the hand movements of a driver. The SVM, implemented on the smartwatch, was used to detect the drowsiness of a driver. Their studies show that smartwatches are capable of analyzing a driver’s driving behavior.

Several studies have been devoted to the problems of driver identification and/or driver authentication. Igarashi et al. [1] built a driving behavior model through the Gaussian mixture model (GMM) on the basis of pressure readings obtained from the accelerator and brake pedals. Similarly, Miyajima et al. [2] used GMMs to model the pedal operation patterns of a driver when following a car for driver identification. Later, Wahab et al. [3] compared GMMs and wavelet transforms in the effectiveness of representing the accelerator and brake pedal pressures for driver identification and authentication. They also compared multilayer perceptrons, fuzzy neural networks, and statistical GMMs in recognition performance, and showed that GMMs are the most effective method. Qian et al. [4] compared three methods for extracting features from the readings of the steering wheel angle, the accelerator and the brake pedals, and applied support vector machines (SVMs) to identify the drivers. Riener and Ferscha [5] installed pressure sensors in the driving seat to capture the driver’s pelvic bone signature. They used the driver’s pelvic bone distance as a biometric feature, and matched the driver’s enrolled patterns by the Euclidean distance. To the best of our knowledge, there has not been any feasibility study on the problem of driver authentication using smartwatches.

In this paper, a smartwatch-based driver authentication mechanism is proposed. A novel GMM-based approach is also developed for building the driving behavior model of a driver. Since a driver usually manipulates the steering wheel differently in different driving maneuvers (e.g., driving straight or turning), we have created a behavioral model for each driving maneuver for each driver. 

The proposed GMM-based approach addresses two weaknesses of the traditional GMM by building a smartwatch-based behavioral model of drivers. First, in the traditional GMM-based approach, the likelihood value of the GMM of an input pattern is often used to determine whether or not the input pattern is drawn from the data distribution modelled by the GMM. However, when, for example, some Gaussian components of the GMM for driver *A* cover all of the Gaussian components of the GMM for driver *B*, it may be difficult to distinguish between drivers *A* and *B* with the likelihood value of the GMM for driver *A*, even though the two GMMs have distinctive Gaussian components. In this paper, to enhance the distinctive Gaussian components of the GMM for each driver, the posterior probabilities of the Gaussian components of GMMs were used instead of the likelihood value of the GMM. Second, in the traditional GMM-based approach [1,2,3], different kinds of features are equally weighted. We found that different kinds of features can be weighted differently to reflect their differing effectiveness for different drivers. To alleviate the two weaknesses of the traditional GMM, we designed two models and combined them through stacked generalization [23] to yield the final driving behavior model.

We established a driving simulation system to collect driving behaviors and recruited 52 participants for the experiment to evaluate the proposed approach. The behaviors of each participant when driving straight, turning left, and turning right were used to construct his/her own models. In the context of driver authentication, if a given participant is selected as the registrant, then all other participants are considered as imposters. The experimental results indicate that the proposed approach can be used to authenticate the driver, with an equal error rate (EER) of 4.62%. Additionally, the proposed approach was tested on 15 participants, who were licensed to drive automobiles, in a real-driving environment, with an EER of 7.86%.

Our main contributions are highlighted below:

(i) A smartwatch-based driver authentication mechanism is presented. We demonstrate that the driver’s hand motion information captured by the built-in sensors of a smartwatch can be used to authenticate the driver.
(ii) A novel GMM-based behavioral modeling approach is also proposed to improve the traditional GMM in modeling driving behavior.
(iii) The experimental results on the data collected from both the simulated and real-traffic environments indicate that the proposed approach is feasible in both environments and more accurate than the traditional GMM.

The remainder of this paper is organized as follows: Section 2 introduces the driving simulation system, the real-traffic environment, and the apparatus used in this study, and Section 3 describes the proposed methodology for driving behavioral modeling and detection. Section 4 discusses the experimental results. Concluding remarks and suggestions for future studies are presented in Section 5.

## 2. Data Collection Environments and Apparatus

### 2.1. The Simulated System

**Figure 1:**
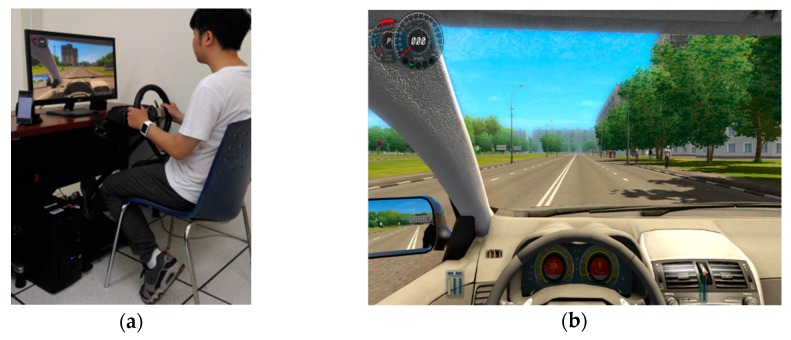
(**a**) Driving simulation system. (**b**) Simulated road scene.

A driving simulation system bearing close resemblance with a real driving system was established to analyze driving behaviors (Figure 1a). The simulation system included a desktop computer, a liquid-crystal display monitor, a simulator-grade wheel, and a pedal unit. The driving simulation software City Car Driving [24] was used to simulate realistic three-dimensional road scenes with dynamic traffic streams (Figure 1b).

Participants were asked to drive using this system as they would drive a real car (their safety was ensured regardless of their driving skill). Although this system did not provide a complete driving experience with fully realistic controls and variable road conditions, it could capture driver behavior in accordance with our criteria for comparing and identifying driving behaviors.

### 2.2. The Real Environment

**Figure 2:**
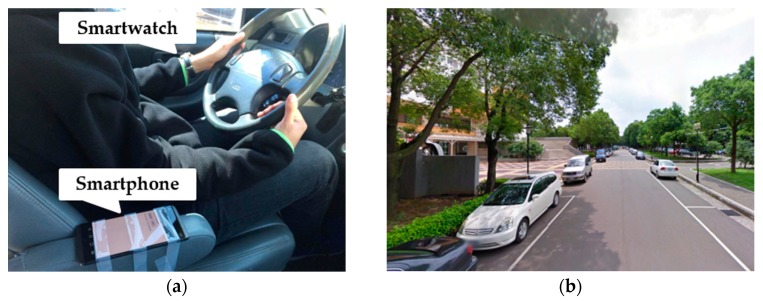
(**a**) Participant in a real vehicle. (**b**) Real-traffic road scene [25].

We also collected the driving behavior data of some participants driving a real vehicle (Honda CR-V) in the campus of National Central University. As shown in Figure 2a, a smartphone was placed in this car beside the driver; the smartphone’s gyroscope readings were used to divide each driver’s driving session into separate segments for different driving maneuvers. The road scene of the campus is shown in Figure 2b.

### 2.3. Apparatus

The Sony SmartWatch 3, the Sony Xperia Z5 Premium (Sony Corp., Tokyo, Japan) and the Logitech G27 racing wheel (Logitech International S.A., Lausanne, Switzerland) [26] were used in the simulating system, and the angle of the steering wheel was acquired through the Logitech Steering Wheel SDK [27]. The LG Watch Urbane and the LG V20 smartphone (LG Electronics Inc., Seoul, South Korea) were used in the real environment; while the sampling rate of the smartwatch’s and smartphone’s built-in sensors were set at 50 Hz in both environments. 

## 3. The Proposed Methodology

**Figure 3:**
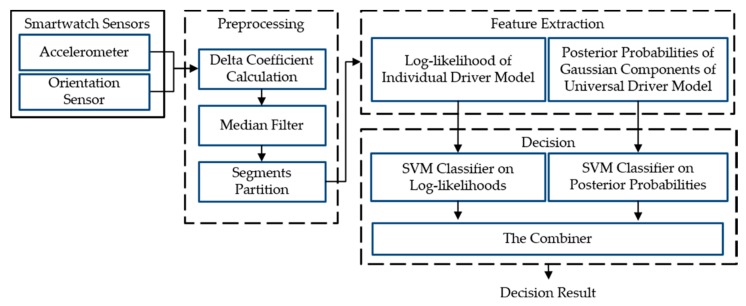
Overview of the proposed smartwatch-based driver authentication mechanism.

The proposed smartwatch-based driver authentication mechanism, as illustrated in Figure 3, has three major steps: (1) the preprocessing; (2) the feature extraction; and (3) the decision.

In our driver authentication mechanism, a driver’s behaviors are directly observed by capturing the data of the smartwatch built-in motion sensors (3-axis accelerometer and 3-axis orientation sensor). In *Preprocessing*, the dynamic data are extracted from the collected motion data, and then the noise of the raw and dynamic data are removed through a median filter. In this study, the entire sensor data sequence is partitioned into segments, so that each segment is focused on a specific operational behavior of an individual. In *Feature Extraction*, two GMM-based driver models are built to extract several features from the preprocessed data. Finally, two types of features are separated to train the classifier model by using SVMs. These two SVMs are combined through stacked generalization to yield a driving behavior model for the driver.

### 3.1. Data Preprocessing

Smartwatch accelerometers and orientation sensors can provide multidimensional data during a single sensor event. Since the z-axis signal of the orientation sensor is the angle to the magnetic north [28], the information from the z-axis is more related to the condition and direction of the road than the driving behavior, and thus is not used in this study. Many studies reported that, compared to static information, the use of dynamic information can improve the accuracy of behavioral biometrics [29,30]. Therefore, for each dimension of the sensor data, the delta (velocity) coefficient, a dynamic data, was adopted. The delta coefficient can be obtained from the following formula:

(1) $$\Delta x\left(t\right)=\frac{\sum_{u=1}^{K}u\left(x\left(t+u\right)-x\left(t-u\right)\right)}{2\sum_{u=1}^{K}u^{2}}$$

where the delta window size *K* was set to 25 on the basis of preliminary experiments [31]. As listed in Table 1, four types of signals were obtained from the accelerometer and orientation sensor. The sensor data collected at time *t* are presented as $x_{t}={\left[\omega_{1;t}^{T},\omega_{2;t}^{T},\omega_{3;t}^{T},\omega_{4;t}^{T}\right]}^{T}\in {\mathbb{R}}^{10}$, where $\omega_{1;t}\in {\mathbb{R}}^{3},\omega_{2;t}\in {\mathbb{R}}^{2},\omega_{3;t}\in {\mathbb{R}}^{3},$ and $\omega_{4;t}\in {\mathbb{R}}^{2}$ are the vectors formed by the three axes of the accelerometer, the x and y axes of the orientation sensor, the delta coefficients with respect to the three axes of the accelerometer, and the delta coefficients with respect to the x and y axes of the orientation sensor, respectively.

**Figure 4:**
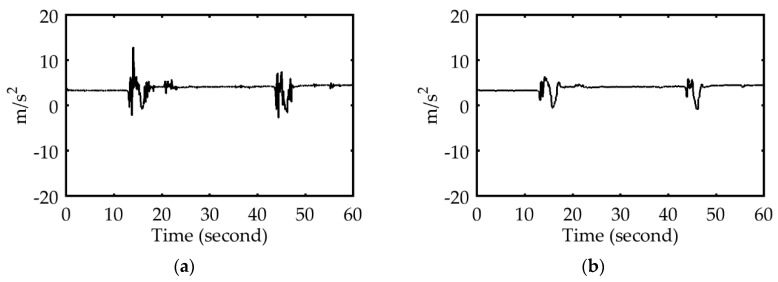
(**a**) X-axis accelerometer signal with noise. (**b**) Median filtered X-axis accelerometer signal.

After removing the noise from the sensor data using a median filter (as shown in Figure 4), the entire sensor data sequence was partitioned into segments by analyzing the steering wheel angle in the simulated environment, and by analyzing the z-axis angular velocity of the smartphone gyroscope in the real-driving environment. Each segment conveyed information regarding the behavior of a driver driving straight, turning left, or turning right, modeling all three driving behaviors.

**Figure 5:**
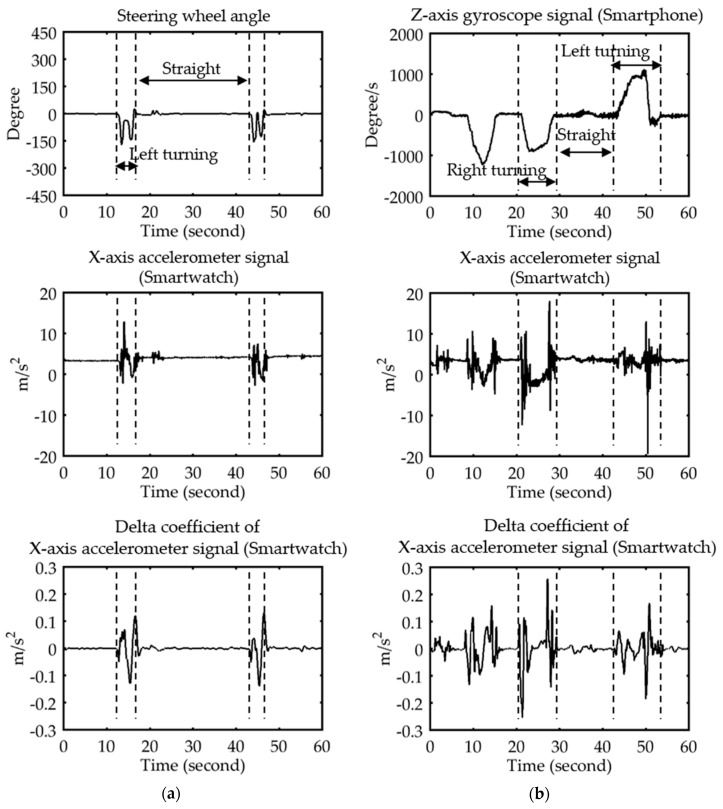
X-axis accelerator and its dynamics for the segment of a single driver. (**a**) Simulated environment. (**b**) Real environment.

In the simulated environment, the threshold value of the steering wheel angle for partitioning the signal was set to ±30°. Figure 5a displays the correlation between the accelerometer and the steering wheel signals at different periods. By contrast, the threshold value of the angular velocity of the smartphone’s gyroscope for partitioning the signal was set to ±10°/s. Figure 5b displays the correlation between the accelerometer and the gyroscope of the smartphone at different periods.

### 3.2. Feature Extraction

The smartwatch sensor data varied according to the driver and the driving scenario. The GMM was used to capture the sensor data distributions for a driver with a specific driving behavior, which is referred to as an individual driver model (IDM). The IDM log-likelihood of the sensor data has already been used for driver recognition [1,2,3,32], where the log-likelihood value of the model is the total sum of each log-likelihood value of the GMMs based on each sensor. In our study, since each of the four features differed in its effectiveness to authenticate genuine drivers, the IDM log-likelihoods for the four features were combined using SVMs in a weighted manner. Furthermore, to enhance the distinctive Gaussian component of a driving behavior, a universal driver model (UDM), which represented the collective behavior of all drivers, was learned by the GMM. Furthermore, SVMs on the posterior probabilities of the Gaussian components of the UDM were used in the study of other driver models. These two base SVMs for each driver were combined through stacked generalization to form each driving behavior model.

The remainder of this section describes two base learners: (1) the base SVM, which was based on the IDM log-likelihoods; (2) the other base SVM, which was based on the posterior probabilities of the Gaussian components of the UDM.

(1) Base Learner 1: SVM Based on the IDM Log-likelihoods

The parameters of the IDM with *M* Gaussian components are denoted by $\theta ={\left\{w_{i},\mu_{i},\Sigma_{i}\right\}}_{i=1}^{M}$. The mixture density of the IDM θ is a weighted sum of *M* Gaussian component densities as follows:

(2) $$P\left(\omega |\theta \right)=\overset{M}{\underset{i=1}{\sum }}w_{i}G(\omega |\mu_{i},\Sigma_{i})$$

where ω is a *D*-dimensional random vector, $w_{i},$ i=1,…,M and $\overset{M}{\underset{i=1}{\sum }}w_{i}=1$ are the mixture weights, and $G\left(\omega |\mu_{i},\Sigma_{i}\right)$, i=1,…,M, is the density function of the multivariate normal distribution,

(3) G(ω|μi,Σi)=1(2π)D2|Σi|12exp{−12(ω−μi)TΣi−1(ω−μi)}

and where $\mu_{i}$ and $\Sigma_{i}$ are the mean vector and covariance matrix for the *i*th component, respectively. The four IDMs, each of which was built on one of the four types of features, were analyzed using the expectation maximization algorithm to construct the driving behavior model for each driver performing each maneuver.

The parameters of the *i*th IDM are denoted by $\theta_{i}={\left\{w_{j;i},\mu_{j;i},\Sigma_{j;i}\right\}}_{j=1}^{M_{i}}$. Let $x_{1},\ldots ,x_{T}$ be a segment of the smartwatch sensor data of a driver. Similar to [1,2,3,32], whether $x_{1},\ldots ,x_{T}$ is generated by the IDMs for a driver performing a specific maneuver can be determined by the log-likelihood of $x_{1},\ldots ,x_{T}$, which is defined as follows:

(4) $$ℒ\left(x_{1},\ldots ,x_{T}|\theta_{1},\ldots ,\theta_{4}\right)=\sum_{i=1}^{4}\frac{1}{T}\sum_{t=1}^{T}\log\left(P\left(\omega_{i;t}|\theta_{i}\right)\right)$$

where $\theta_{1},\ldots ,\theta_{4}$ are the parameters of the IDMs for the four features. In this study, the following linear SVM, which is the first base SVM, $S_{IDM}\left({\left[\begin{array}{ccc} \frac{1}{T}\sum_{t=1}^{T}\log\left(P\left(\omega_{1;t}|\theta_{1}\right)\right) & \ldots & \frac{1}{T}\sum_{t=1}^{T}\log\left(P\left(\omega_{4;t}|\theta_{4}\right)\right) \end{array}\right]}^{T}\right)$ was estimated to weigh the four IDM log-likelihoods differently. The SVM $S_{IDM}$ also outputted an estimate of the posterior probability so that the input feature vector was a positive sample.

(2) Base Learner 2: SVM Based on Posterior Probabilities of Gaussian Components of the UDM

**Figure 6:**
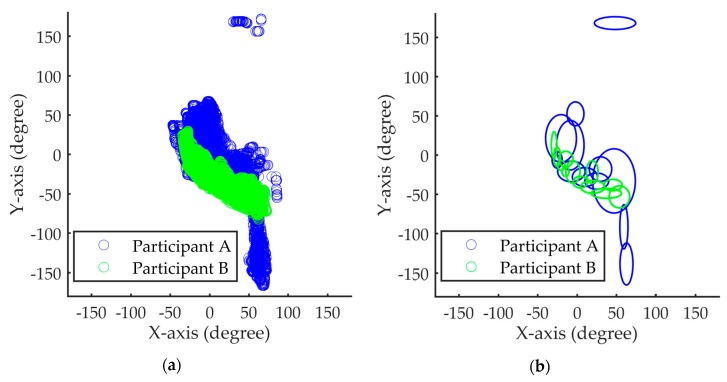
Orientation sensor signals of the driving behaviors from two different participants A and B: (**a**) data distribution signals; (**b**) components of two Gaussian mixture model (GMMs).

The limitation of the IDM can be explained by the following example. As shown in Figure 6, the Gaussian component set of participant B is a subset of that of participant A. Given a test sample (either A or B), the likelihood of the test sample is calculated by the summation of the responses of all the Gaussian components (or the log-likelihood) of the IDM of participant A. The test sample is classified as Class “A” if the likelihood value is higher than a preset threshold. The likelihood of B’s sample (from A’s IDM) is always high since B’s Gaussian components are also A’s. Therefore, participant A’s IDM always misclassifies B’s samples as A.

The proposed method takes two steps to alleviate the current limitation. In addition to the IDM models for each participant, we have created a UDM based on the data of all participants. This UDM represents the behavioral patterns of all participants; therefore, the Gaussian component sets of both A and B are subsets of this UDM. Furthermore, we have built a SVM-based classifier that uses the individual response of each component as an independent feature. This classifier is able to distinguish Participant B from A since B’s Gaussian components are only a subset of A’s, but are not identical to A’s.

To use the distinctive Gaussian components of driving behaviors, four UDMs, each constructed on the basis of one of the four features, were estimated to build a GMM for the collective behavior in a specific driving scenario. Subsequently, $x_{t}$ was mapped to vector $f_{t}$ in a new d-dimensional space by using the formula

(5) $$f_{t}={\left[f_{1;1;t},f_{2;1;t},\ldots ,f_{M_{1};1;t},\ldots ,f_{1;4;t},\ldots f_{M_{4};4;t}\right]}^{T}$$

where $d=\sum_{i=1}^{4}M_{i}$ is the total number of Gaussian components of the four UDMs and $f_{j;i;t}$ is the posterior probability that $\omega_{i;t}$ is generated by the *j*th Gaussian component of the *i*th UDM:

(6) $$f_{j;i;t}=\frac{w_{j;i}G\left(\omega_{i;t}|\mu_{j;i},\Sigma_{j;i}\right)}{\sum_{k=1}^{M_{i}}w_{k;i}G\left(\omega_{i;t}|\mu_{k;i},\Sigma_{k;i}\right)}$$

In this *d*-dimensional space, a linear SVM $S_{UDM}\left(\frac{1}{T}\sum_{t=1}^{T}f_{t}\right)$, which is the second base SVM, was calculated. This SVM $S_{UDM}$ also outputted the posterior probability that the input feature vector was a positive sample.

### 3.3. Proposed Driving Behavior Model

**Figure 7:**
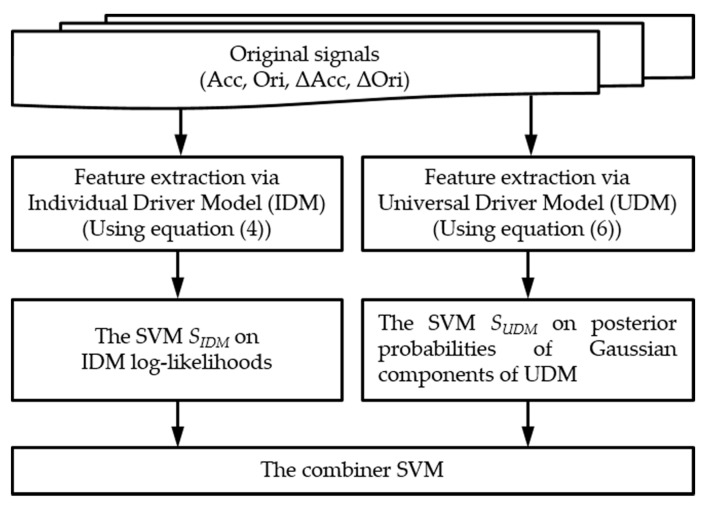
Proposed driving behavior model for a driver in a specific driving scenario.

Two modalities based on linear SVMs—$S_{IDM}$ and $S_{UDM}$— were trained on the different feature vectors. As shown in Figure 7, another combiner SVM was used to combine $S_{IDM}$ and $S_{UDM}$. Stacked generalization can be used to illustrate this combination framework. The key idea is to evaluate a meta-learner based on the outputs of multiple base-learners. Some researchers [33,34] have demonstrated that the base-learners and meta-learner can use the same learning algorithm to handle multimodalities. In the present study, the combiner SVM also outputs the posterior probability that the input is a positive sample. Additionally, these driving behavior models for a driver can be applied in several driving scenarios to determine if the driver drives as usual in these driving scenarios. To this end, the average output of the driving behavior models for a driver can be used in these different driving scenarios. In the present work, we built three driving behavior models for a driver in three specific driving scenarios: driving straight, turning left, and turning right.

## 4. Experiments and Discussion

Three experiments were conducted to evaluate the proposed approach. The purposes of the experiments were as follows: (1) to analyze the number of Gaussian components of the GMM required for the proposed approach; (2) to evaluate the accuracy of the proposed approach for driver authentication in the simulated environment; and (3) to evaluate the accuracy of the proposed approach in the real-traffic environment.

The implementations of the traditional GMM approach and the proposed approach were as follows:

- GMM: The traditional GMM technique (using function (4)) is hereafter referred to as the GMM approach. The implementation of GMMs in the statistics and machine learning toolbox of MATLAB was employed for performance comparison.
- Stacking: The proposed approach (the combiner SVM as shown in Figure 7) is hereafter referred to as the stacking approach. It was implemented in MATLAB 2017a with LIBSVM [35].

All analyses were conducted on a personal computer (Predator G3610, Acer Inc., New Taipei City, Taiwan) with an Intel Core i7-2600 CPU (Intel Corp., Santa Clara, CA, USA) and 16 gigabytes of RAM, and run in the Windows 7 operating system (Microsoft Corp., Washington, DC, USA).

### 4.1. Experimental Setups

(1) Data Acquisition

Fifty-two volunteers, including 27 licensed drivers and 25 unlicensed persons, were recruited from various departments of National Central University for the experiment, with a mean age of 24 ± 2 years. All subjects gave their informed consent for inclusion before they participated in the study. The study was conducted in accordance with the Declaration of Helsinki, and the protocol was approved by the Ethics Committee of National Taiwan University (201802ES007). To ensure that these participants were familiar with the simulated system, they were trained until they could maintain normal driving (driving in oncoming traffic lanes and weaving in and out of traffic were prohibited). We also ensured that every participant maintained a good mental state and did not drink before data collection. They were asked to wear a smartwatch on their left hand and proceeded with their most comfortable driving behavior to operate the steering wheel. The participants in the real environment also adhered to the same requirement.

**Figure 8:**
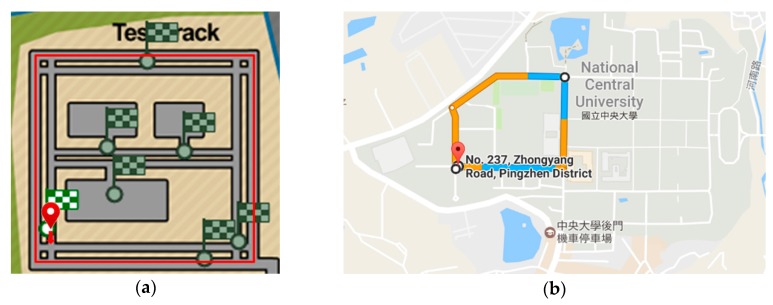
(**a**) The route in the simulated environment. (**b**) The real-traffic route.

Figure 8a shows the route of the simulated environment. Each participant was asked to drive the route under similar traffic conditions clockwise 40 times, and counterclockwise 40 times to collect the driving behavior of the participant when turning right and left. In total, each participant undertook 80 driving sessions; each session lasted an average of 180 seconds (s) (9000 data points in average). The 80 driving sessions were completed in eight rounds of experiments over the course of three weeks. In total, 14,832 segments of driving straight, 8434 segments of turning left, and 12,168 segments of turning right were collected. Each segment was regarded as a driving behavior sample.

Fifteen participants (of 52 volunteers) were also involved in the data collection in the real-traffic environment. They all had driver’s licenses and had at least two years of driving experience. As shown in Figure 8b, the route for this experiment has five turns and is approximately 1.77 km long. Each participant was asked to drive the route clockwise and counterclockwise so that their driving behavior when turning right and left, respectively, could be collected. Each participant was required to be familiar with the road of the campus prior to data collection. In consideration of the risk of real-traffic roads, data acquisition was conducted only in the daytime between 9 a.m. and 5 p.m., and if there were sunny weather conditions. Every participant undertook 20–25 driving sessions, with each session lasting for 345 s on average (17,250 data points on average). All the driving sessions of a participant were collected in four rounds of experiments over two weeks. In total, 3172 segments of driving straight, 2093 segments of turning left, and 1428 segments of turning right were collected.

(2) Evaluation and Performance Indices

To estimate the performance indices for each participant, the driving behavior of a given participant was regarded as the registrant’s behavior, while the driving behavior of the other participants was regarded as the imposter’s behavior. For each participant, 51 pairs of training and test sets were produced by the leave-one-person-out strategy. The training set comprised 55 segments of the registrant’s driving behavior (as positive samples) and 80 segments of the imposter’s driving behavior (as negative samples). The test set comprised another 20 segments of the registrant’s driving behavior and 20 segments of the imposter’s driving behavior. Participants who provided negative samples in the training set contributed no samples to the test set.

The false acceptance rate (FAR), false rejection rate (FRR), detection error trade-off (DET) curve, area under curve (AUC), and EER were used as performance indices for all the experiments. The FAR is defined as the percentage of the imposter’s behaviors that was wrongly recognized as the registrant’s behaviors, and the FRR is the percentage of the registrant’s behaviors that was wrongly recognized as the imposter’s behaviors. Both FAR and FRR depend on the threshold limit used in the decision-making process. In the detection process, the DET curve was used to illustrate the relationship between the FAR and FRR by varying the threshold limit [36]. The AUC of the DET curve is proportional to the product of the FAR and the FRR. Minimizing the AUC of the DET curve is equivalent to reducing either one of the error types or both [37]. The EER is the value where the FAR and FRR become equal by adjusting the threshold. The EER performance measure rarely corresponds to a realistic operating point. However, it is a relatively popular measure of the ability of a system to distinguish between the two categories [38].

The models obtained for each driving maneuver were annotated with an “S” (for driving straight), “L” (for turning left), or “R” (for turning right), and the “stacking S + L + R approach” referred to the stacking approach that utilized the three segments, with each annotation representing one of the three maneuvers.

### 4.2. Experimental Results

(1) Experiment 1: Analysis of the Number of Gaussian Components

**Figure 9:**
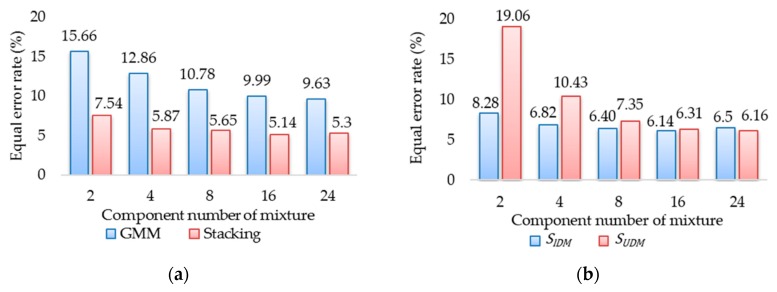
Equal error rate (EER) with respect to various numbers of Gaussian components: (**a**) GMM and Stacking; (**b**) two base SVMs $S_{IDM}$ and $S_{UDM}$.

The number of Gaussian components required for the GMM was analyzed from 15 participants in the simulated environment as follows. Figure 9a presents the EER, while Table 2 presents the training time of the GMM and the stacking approaches in the S + L + R driving scenario with respect to 2, 4, 8, 16, and 24 Gaussian components. The training time was proportional to the number of Gaussian components, and the EER decreased as the number of Gaussian components increased from 8 to 24. Figure 9b provides the EER of the two base SVMs $S_{IDM}$ and $S_{UDM}$. Notably, they did not require the same number of Gaussian components. Figure 9b and Table 2 show that when the number of GMM components of $S_{IDM}$ increased from 4 to 8, the accuracy of $S_{IDM}$ improved by 6.17% but the training time increased by 216%. The results also show that the accuracy of $S_{UDM}$ improved by 14.15% and the training time increased by 54.63% when the number of GMM components of $S_{UDM}$ increased from 8 to 16. After evaluating the trade-off between the EER and the training time, the number of GMM components was set to 4 for the IDM and 16 for the UDM. In this parameter setting, the average training time and the average testing time of the stacking S+L+R approach were 172 s and 0.04 s, respectively. Additionally, since the stacking approach is more computationally complicated, it was slower than the IDM approach. However, the computational cost of the stacking approach was acceptable.

(2) Experiment 2: Performance Evaluation of Driver Authentication in the Simulated Environment

**Figure 10:**
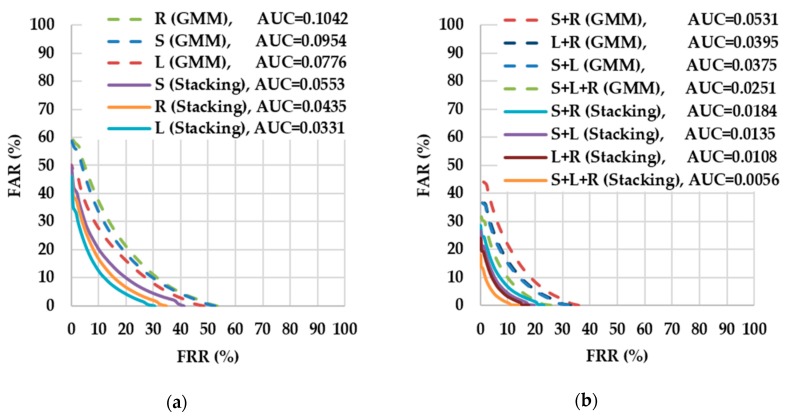
Detection error trade-off (DET) curves for different numbers of driving behavior models using the GMM and stacking approaches: (**a**) single driving scenario; (**b**) multiple driving scenario.

Figure 10 displays the DET curves of the GMM and the stacking approaches. Figure 10a reveals that the stacking approach was more accurate than the GMM approach in single driving scenarios. In addition, as presented in Figure 10b, the accuracy of the stacking and the GMM approaches improved after multiple driving scenarios, with the stacking approach remaining more accurate than the GMM approach in multiple driving scenarios. Table 3 demonstrates that the stacking approach was at least 4% more accurate than the GMM approach when considering the EERs of each approach. Therefore, the experimental results indicated that the proposed approach outperformed the GMM approach, and thus supports the proposed approach as a feasible method for verifying drivers in the simulated environment.

(3) Experiment 3: Performance Evaluation of Driver Authentication in a Real-Traffic Environment

Fifteen participants were involved in experiments conducted in a real-traffic environment. As Table 4 indicates, the stacking approach was more accurate than the GMM approach, and the EER of the proposed approach for the S + L + R driving scenario was 7.86%. Table 4 also compares the experimental results of these participants in the real-traffic environment compared to the simulated environment. According to the 2-sample *t* test, the stacking approach attained similar EERs for the S and R driving scenarios in the real-traffic (*p* = 0.094) and simulated (*p* = 0.438) environments. However, the stacking approach was less accurate for the L driving scenario in the real-traffic environment (*p* = $1.064\times 10^{-8}$). One possible reason may be that the participants made left turns more carefully in the real-traffic environment, and thus, their left-turning behavior was not as distinguishable as in the simulated environment. Another possibility is that the real-traffic environment had only one lane of traffic whereas the simulated environment had two. Therefore, driving maneuvers in the simulated environment may have been easier for participants in the simulated environment compared to the real-traffic environment. Nevertheless, the experimental results indicated that the proposed approach holds feasibility in a real-traffic environment.

### 4.3. Discussion

Drivers’ physical and mental states might affect their driving behaviors. Additionally, in real-life driving, there are driving scenarios, such as parking and backing a car, that were not considered in the experiment. In this study, driving scenarios and drivers’ physical and mental states were controlled to reduce the complexity of the experiments. However, in Experiments 2 and 3, we found that the accuracy of driver authentication could be improved if more driving maneuvers were used. For example, the S + L + R driving scenario would have resulted in a higher accuracy than the S + L and S driving scenarios. This was probably because more driving maneuvers provided more information about the driver. These issues are worthy of further investigation.

Table 5 gives a summary of the accuracy of several authentication mechanisms based on a driver’s behavioral characteristics, and shows that our approach is a promising means of driver authentication. Table 5 does not include other studies on smartwatch-based driver authentication because, to the best of our knowledge, these studies are scarce.

## 5. Conclusions

To conclude, the driving behavior of a driver was analyzed from his/her use of a steering wheel. Data from the built-in sensors of a smartwatch attached to the driver’s left hand were used for the analysis. The driving behavior was also modeled by proposing a GMM-based modeling approach. To demonstrate the feasibility of the proposed method, we created an experimental system that analyzed driving behavior using two built-in sensors of a smartwatch. The experimental results indicated that the proposed approach had EERs of 4.62% in a simulated environment and 7.86% in a real-traffic environment, confirming the feasibility of this approach.

The proposed modeling approach has potential for other applications, such as detecting whether drivers maintain normal/habitual behaviors to ensure driving safety. We also believe that the proposed approach can be applied on other kinds of sensing devices. In future works, we intend to investigate the possibility of implementing this authentication mechanism on a smartwatch, and apply the proposed modeling approach to more applications.

## Figures and Tables

**Table 1:** Four signals derived from the built-in smartwatch sensors.

Signal Type	Description
Acc	The three-dimensional signal of the accelerometer
Ori	The two-dimensional signal of the orientation sensor
ΔAcc	The three delta coefficients with respect to the three-dimensional signal of the accelerometer
ΔOri	The two delta coefficients with respect to the two-dimensional signal of the orientation sensor

**Table 2:** Average training time with respect to the number of Gaussian components.

Time (seconds)	GMM	Stacking	*S_IDM_*	*S_UDM_*
2 Components	1.82	13.29	3.07	10.21
4 Components	7.58	38.29	8.98	29.33
8 Components	26.86	134.68	28.41	106.04
16 Components	43.65	209.89	45.46	163.97
24 Components	78.66	300.58	80.74	219.21

**Table 3:** EERs for the GMM and stacking approaches for various driving scenarios.

Driving Scenario	Simulated Environment	
	GMM	Stacking
S	19.39%	14.65%
L	18.02%	11.07%
R	20.53%	12.88%
S+L	12.14%	7.07%
S+R	14.48%	8.35%
L+R	12.47%	6.33%
S+L+R	9.86%	4.62%

**Table 4:** EERs of the GMM and stacking approaches in real-traffic and simulated environments.

Driving Scenario	Real-Traffic Environment		Simulated Environment	
	GMM	Stacking	GMM	Stacking
S	20.93%	16.40%	25.07%	18.38%
L	29.10%	18.33%	17.46%	10.91%
R	24.74%	15.33%	24.15%	15.14%
S+L	18.34%	11.46%	14.44%	8.20%
S+R	17.41%	10.52%	20.30%	11.41%
L+R	20.52%	10.82%	13.70	6.90%
S+L+R	15.67%	7.86%	12.86%	6.07%

**Table 5:** Summary of the EERs of user authentication using various behavioral characteristics.

Behavioral Characteristic	Performance (%)	Participants
Car driving signals [3]	EER = 3.44 to 5.02	30
Gait/Stride [39]	EER = 5 to 6	21
Keystroke dynamics [40]	FAR = 0.01; FRR = 4	154
Mouse dynamics [41]	FAR = 2.465; FRR = 2.461	22
Touch Gestures [42]	EER = 2.35 to 2.99	51
Our proposed approach	EER = 4.62	52
